# *Mycobacterium tuberculosis* Ser/Thr Protein Kinase B Mediates an Oxygen-Dependent Replication Switch

**DOI:** 10.1371/journal.pbio.1001746

**Published:** 2014-01-07

**Authors:** Corrie Ortega, Reiling Liao, Lindsey N. Anderson, Tige Rustad, Anja R. Ollodart, Aaron T. Wright, David R. Sherman, Christoph Grundner

**Affiliations:** 1 Seattle Biomedical Research Institute, Seattle, Washington, United States of America; 2 Department of Global Health, University of Washington, Seattle, Washington, United States of America; 3 Biological Sciences Division, Pacific Northwest National Laboratory, Richland, Washington, United States of America; Harvard University, United States of America

## Abstract

The majority of *Mycobacterium tuberculosis* (*Mtb*) infections are clinically latent, characterized by drug tolerance and little or no bacterial replication. Low oxygen tension is a major host factor inducing bacteriostasis, but the molecular mechanisms driving oxygen-dependent replication are poorly understood. Here, we tested the role of serine/threonine phosphorylation in the *Mtb* response to altered oxygen status, using an *in vitro* model of latency (hypoxia) and reactivation (reaeration). Broad kinase inhibition compromised survival of *Mtb* in reaeration. Activity-based protein profiling and genetic mutation identified PknB as the kinase critical for surviving hypoxia. *Mtb* replication was highly sensitive to changes in PknB levels in aerated culture, and even more so in hypoxia. A mutant overexpressing PknB specifically in hypoxia showed a 10-fold loss in viability and gross morphological defects in low oxygen conditions. In contrast, chemically reducing PknB activity during hypoxia specifically compromised resumption of growth during reaeration. These data support a model in which PknB activity is reduced to achieve bacteriostasis, and elevated when replication resumes. Together, these data show that phosphosignaling controls replicative transitions associated with latency and reactivation, that PknB is a major regulator of these transitions, and that PknB could provide a highly vulnerable therapeutic target at every step of the *Mtb* life cycle—active disease, latency, and reactivation.

## Abstract 2

*Mtb* growth and replication are sensitive to altered levels of the Ser/Thr kinase PknB, and this sensitivity increased under hypoxic conditions. Thus, PknB is a critical regulator of the oxygen-dependent replication switch of *Mycobacterium tuberculosis*.

## Author Summary

Exposure to *Mycobacterium tuberculosis* (*Mtb*) can result in a latent form of tuberculosis (TB) infection, which can then reactivate and progress to active disease. With 1.8 billion infected persons and no tools to predict who will proceed to active disease, latency and reactivation are among the major challenges of TB treatment and control. Oxygen is one of the environmental triggers affecting the balance between latency and reactivation. Normal oxygen levels promote exponential bacterial growth in culture, but low oxygen levels (hypoxia) inhibit such growth, inducing reversible bacteriostasis. How *Mtb* regulates this oxygen-dependent replication switch, however, is still unknown. Here we tested the role of serine/threonine protein kinases—signaling molecules that transmit environmental cues into cellular responses—in this process. We found that kinase inhibition led to a bacterial survival defect and we specifically identified the PknB kinase as a critical regulator of the oxygen-dependent replication switch. *Mtb* growth was sensitive to elevated levels of PknB and this sensitivity increased in hypoxia. Inhibition of PknB activity led to defects in *Mtb* replication. These data show that signaling through PknB modulates the growth and replication state of *Mtb* in response to oxygen, suggesting that PknB could be a drug target by which to control both the active replicating and latent nonreplicating forms of *Mtb*.

## Introduction

*Mtb* can survive for decades in an asymptomatic, clinically latent state before reactivating to active disease. Latent *Mtb* infection is estimated to affect 30% of the world's population, providing a large reservoir for reactivation to active disease [1]. Reactivation occurs in 2%–10% of latently infected individuals during their lifetimes [2],[3], and in individuals with HIV co-infection, the rate of reactivation rises >20-fold [4].

The environmental factors that drive latency transitions and the *Mtb* signals associated with latency and reactivation are poorly characterized. Of these, oxygen tension may be the best understood and is likely to be a major determinant [5]. Oxygen levels are closely linked to mycobacterial growth rate *in vitro* and *in vivo* [6]–[8]. Oxygen tension in granulomas is low [9],[10]; conversely, reactivation from latency occurs mostly in the most oxygen-rich sites of the lung, the upper lobes [11]. Together, these data indicate that hypoxia shapes latency and reactivation during human infection. Latency also has a profound impact on the treatment of TB and the emergence of drug resistance. Several studies suggest that the environment in granulomas promotes bacteriostasis and phenotypic drug resistance [12]–[15].

Reversible protein Ser/Thr phosphorylation is a central mechanism for sensing and responding to external cues. While the classical two-component systems are underrepresented in *Mtb* compared to other bacteria with similar genome size, the “eukaryotic like” serine/threonine protein kinase (STPK) family is expanded, with 11 members [4],[16]. Recent studies implicate bacterial STPKs in development, stress response, and host–pathogen interactions [17], processes that are also at the center of *Mtb* latency and reactivation. A role in cell wall generation and growth has been suggested for PknA and PknB in *M. smegmatis* and *M. bovis BCG* [18], and for PknB in *Mtb* [19]. A recent study provided a global survey of *Mtb* phosphoproteins [20] and identified hundreds of Ser/Thr phosphorylation sites, indicating broad regulation of mycobacterial physiology by Ser/Thr phosphorylation. Importantly, many phosphorylation events are specific to different growth conditions encountered during infection such as low pH, nitric oxide exposure, and hypoxia, further supporting the idea that Ser/Thr phosphorylation is a major signaling mechanism in response to changing environments.

Here, we used a defined *in vitro* hypoxia model to test the role of phosphosignaling in the replicative states that together define the *Mtb* life cycle: active disease (aerated growth), latency (hypoxia), and reactivation (reaeration). We identified PknB as a major regulator of the oxygen-dependent replication switch. PknB levels were reduced in hypoxia and restored in reaeration, suggesting a role for PknB in transducing growth and replication signals. Consistent with this finding, artificially elevated PknB levels during hypoxia led to killing of *Mtb*, while reduced PknB activity affected regrowth upon reaeration. These data provide a link between phosphosignaling and oxygen-dependent replication in *Mtb*, and suggest therapeutic strategies for targeting latent and reactivating infection.

## Results

### STPK Inhibition Compromises Survival in Reaeration

**Figure 1:**
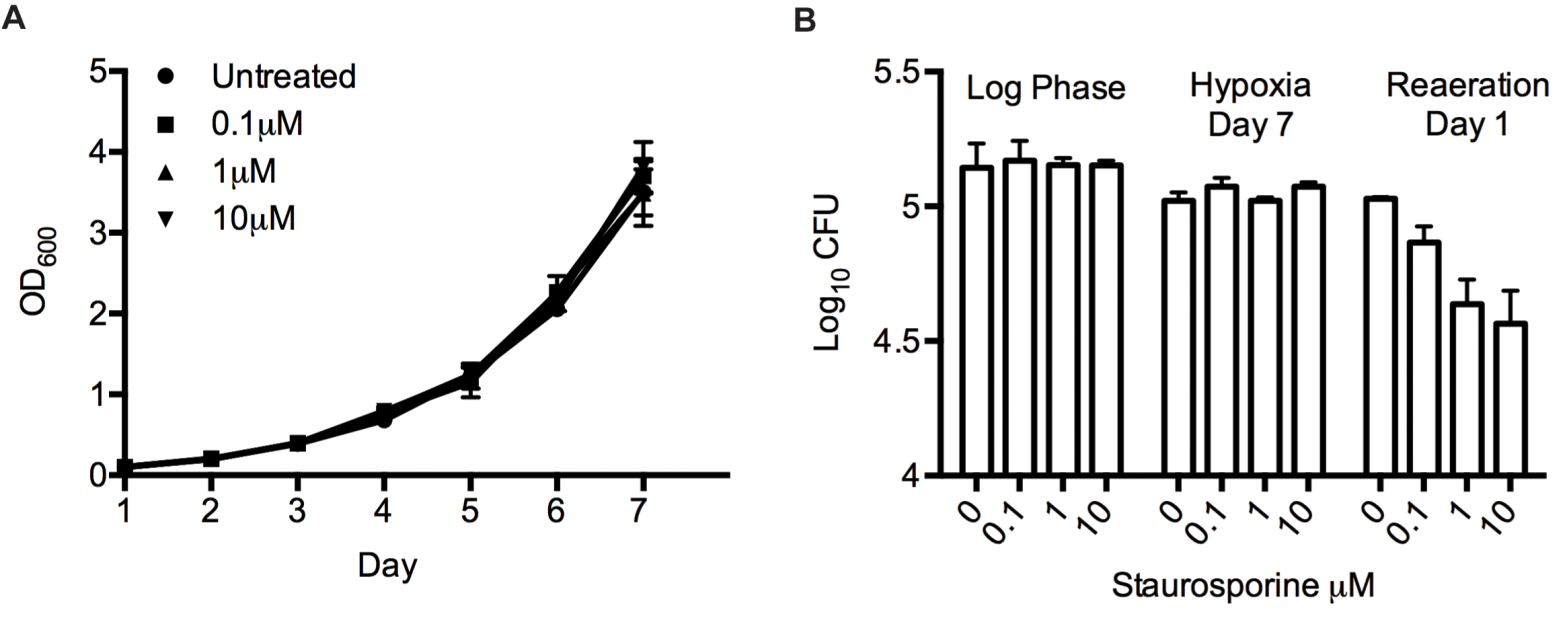
Broad STPK inhibition compromises *Mtb* growth in reaeration. (A) Wild-type *Mtb* in aerated log phase culture was treated with sub-MIC concentrations (0.1, 1, and 10 µM) of staurosporine, and growth was measured over 7 d. (B) Viability was determined on day 0, day 7 of hypoxia, and day 1 of reaeration by plating for CFU analysis. Error bars represent standard deviation.

To determine the role of STPKs in oxygen-dependent replication, we used an *in vitro* model of hypoxia and reaeration [5] in combination with chemical STPK inhibition. In this system, *Mtb* in midlog phase is placed in hypoxia (0.2% oxygen) for 7 d to induce bacteriostasis, then returned to normoxia for resumption of growth [21],[22]. To test whether *Mtb* STPKs regulate oxygen-dependent replication, we treated bacteria with the broad kinase inhibitor staurosporine. Staurosporine inhibits *Mtb* growth with a minimal inhibitory concentration between 25 and 50 µM [23]. To test effects of staurosporine on oxygen-dependent replication, we first determined staurosporine concentrations that allow for unaltered aerated growth. H37Rv cultures treated with 10 µM staurosporine or less displayed growth kinetics similar to untreated *Mtb* during aerated growth (Figure 1A). We next tested the effect of 10 µM staurosporine on *Mtb* during hypoxia and reaeration. Hypoxia led to rapid growth arrest of untreated *Mtb*, and regrowth after reaeration as previously observed [5],[24],[25]. Similarly, staurosporine-treated *Mtb* was not compromised in hypoxia; however, staurosporine-treated *Mtb* lost viability upon reaeration in a dose-dependent manner. Concentrations as low as 0.1 µM staurosporine reduced survival as determined by colony forming unit (CFU) assay (Figure 1B) and a direct readout of ATP measuring bacterial viability (Figure S1). The 10 µM dose of staurosporine, while not affecting aerated cultures, resulted in a ∼5-fold decrease in CFU and ATP levels upon reaeration. These data suggested that STPK inhibition is tolerated during hypoxia, but that one or more STPKs have a causal role in resuming growth and division of *Mtb* in reaeration.

### PknF, PknD, and PknB Are the Primary Targets of Staurosporine

**Figure 2:**
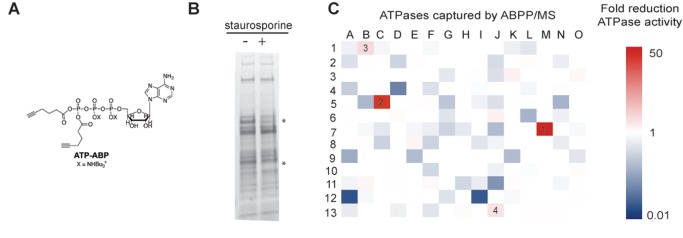
Competitive ABPP identifies PknB, D, and F as staurosporine targets. (A) Structure of the ATP activity-based probe. (B) Grey scale image of *Mtb* lysate fluorescently labeled following ABPP. Detergent-solubilized *Mtb* proteome was treated with staurosporine at 25-fold molar excess over ATP-ABP before labeling with ATP-ABP at subsaturating concentrations and analysis by SDS-PAGE. Probe shows labeling of ATPases and competition of staurosporine with binding to some ATPases (asterisks). (C) Heatmap representation of MS analysis of competitive ABPP to identify staurosporine targets. ATPase abundance was measured by quantitative, AMT tag approach mass spectrometry. Each square represents one *Mtb* H37Rv protein, ordered by Rv number from left to right. Color-coding represents the average fold-reduction of protein quantity in staurosporine-treated versus untreated samples from six technical replicates. Reduced ATP-ABPP probe binding in the presence of staurosporine identifies PknF (1), PknE (2), and PknB (3) as the primary targets of staurosporine.

In mammalian cells, staurosporine is known to act on numerous STPKs [26]. This binding promiscuity limits the use of staurosporine for defining the contribution of individual STPKs to the reaeration phenotype; however, based on the binding properties of this compound in the human proteome [26], we expected that only a subset of *Mtb* STPKs binds staurosporine. To identify these targets in *Mtb*, we used a competitive activity-based protein profiling (ABPP) approach (Figure 2) [27]. We incubated detergent-solubilized *Mtb* proteome with staurosporine at 25-fold molar excess over an ATP activity-based probe (ATP-ABP) (Figure 2A) [28],[29]. Then, we labeled staurosporine-treated and -untreated samples with ATP-ABP at subsaturating concentrations. Using a click-chemistry enabled biotin tag, we purified ATP-ABP-labeled proteins from control and staurosporine-treated samples. By quantitative accurate mass and time (AMT) tag mass spectrometry [30], we quantitated the reduction in ATP-ABP binding of individual ATPases in the presence of staurosporine. ATP-ABP labeled ∼200 ATPases (Figure 2B,C and Data S1) [28]. The greatest reduction of ATP-ABP binding in the presence of staurosporine was detected for three STPKs: PknB, PknD, and PknF. ATP-ABP labeling of PknF by staurosporine was strongest. No PknF peptides were detected by mass spectrometry in the staurosporine-treated samples, and the reduction of binding was arbitrarily set to 50-fold. ATP-ABP labeling of PknD was reduced 40-fold, and PknB labeling was reduced 10-fold (Figure 2C). Other staurosporine targets that showed reduced ATP-ABP labeling in the presence of staurosporine included the nonessential possible fatty acid synthase Rv3720 with almost 8-fold reduction of signal, while all other proteins showed <5-fold reduction of ATP-ABP binding (Data S1). Proteins showing less than 5-fold reduction of ATP-ABP binding are unlikely to bind staurosporine during the hypoxia experiment because the staurosporine concentration used in hypoxia was 50-fold lower than that used in the competitive ABPP screen. These ABPP data define the staurosporine binding profile in *Mtb*, identify the STPKs PknB, D, and F as its main targets, and link PknB, D, and F to the survival defects in staurosporine-treated reaerated cultures.

### PknD, PknF, and PknH Are Not Required for Oxygen-Dependent Replication

To assess the individual roles of PknD and PknF in hypoxia adaptations, we obtained transposon mutants of *pknD* (*tn:pknD*) and *pknF* (*tn:pknF*) [31]. Insertions map to nucleotide positions 89 and 380 in *tn:pknD* and *tn:pknF*, respectively, truncating the N-terminal kinase domains and rendering them inactive. The *tn:pknD* and *tn:pknF* mutants grew similarly to WT in log phase (Figure S2A). To test if *pknD* and *pknF* are conditionally required in hypoxia and/or reaeration, we exposed *tn:pknD* and *tn:pknF* mutant strains to hypoxia and reaeration and assayed viability. Similar to wild type, the mutant strains ceased to replicate in hypoxia and returned to exponential growth upon reaeration with no loss in viability (Figure S2B,C). These data suggest that PknD and PknF are not required for surviving hypoxia or growth upon reaeration, although we cannot rule out the possibility that loss of STPK activity in the two single mutants is compensated for by one or more of the other STPKs. A previous study showed that PknH, which was not covered in our screen, activates the initial hypoxic response regulator DosR [32]. To test if PknH affects growth in response to hypoxia, we tested a *tn:pknH* mutant in the hypoxia time course. The *tn:pknH* mutant showed no growth defects in hypoxia or reaeration, consistent with a limited role of DosR in hypoxia [21]. Since individually PknD, PknF, and PknH are not required for hypoxic survival, we next assessed the contribution of PknB.

### Chemical Inhibition of PknB Affects Regrowth

**Figure 3:**
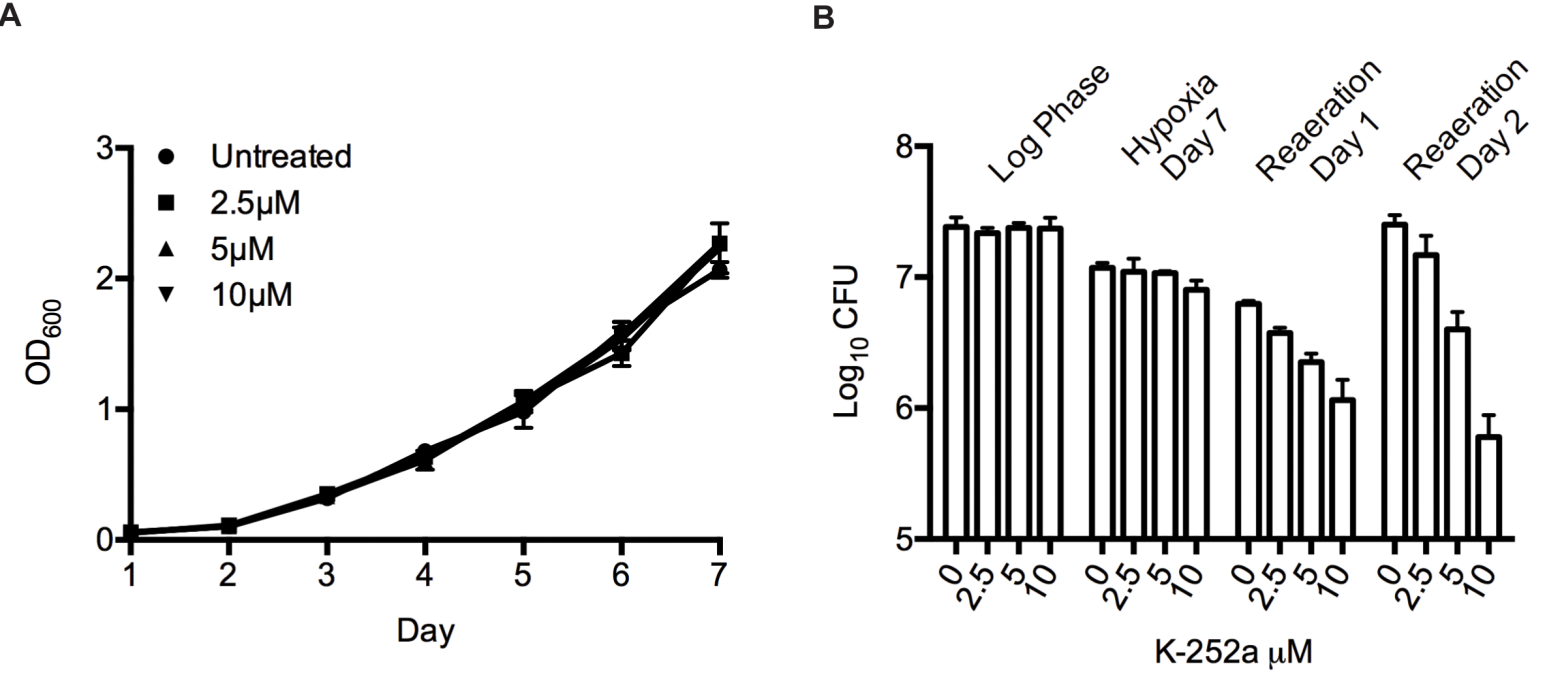
Chemical inhibition of PknB compromises viability in reaeration. (A) Wild-type *Mtb* in log phase was treated with sub-MIC concentrations of K252a, and growth in aerated culture was measured for 7 d. (B) Viability during a hypoxia time course was determined on day 0, hypoxia day 7, and reaeration day 1 and 2 by CFU analysis. Error bars represent standard deviation.

To determine the individual contribution of PknB, we first adopted a chemical inhibition strategy using the inhibitor K252a, which binds PknB with ∼2-fold higher affinity than staurosporine [23]. We determined the highest K252a concentration that did not affect aerated *Mtb* growth to be 10 µM (Figure 3A). We then exposed an aerated culture of *Mtb* to different concentrations of K252a immediately before induction of hypoxia. Bacterial survival after 7 d of hypoxia and after 1 and 2 d of reaeration was measured by CFU assay, and by ATP levels after 2 d of reaeration (Figure 3B, Figure S3). Similar to our experiments with staurosporine, K252a showed only small effects on *Mtb* survival in hypoxia. However, K252a exposure at the start of hypoxia caused a dose-dependent reduction in viability upon reaeration when compared to untreated *Mtb*. K252a at 10 µM reduced viability upon reaeration 5-fold on day 1 of reaeration and almost 10-fold on day 2 of reaeration (Figure 3B, Figure S3). Because we cannot rule out off-target effects of K252a, we next sought to test the role of PknB in hypoxia and reaeration by a genetic approach.

### PknB Levels Are Regulated in Response to Hypoxia

**Figure 4:**
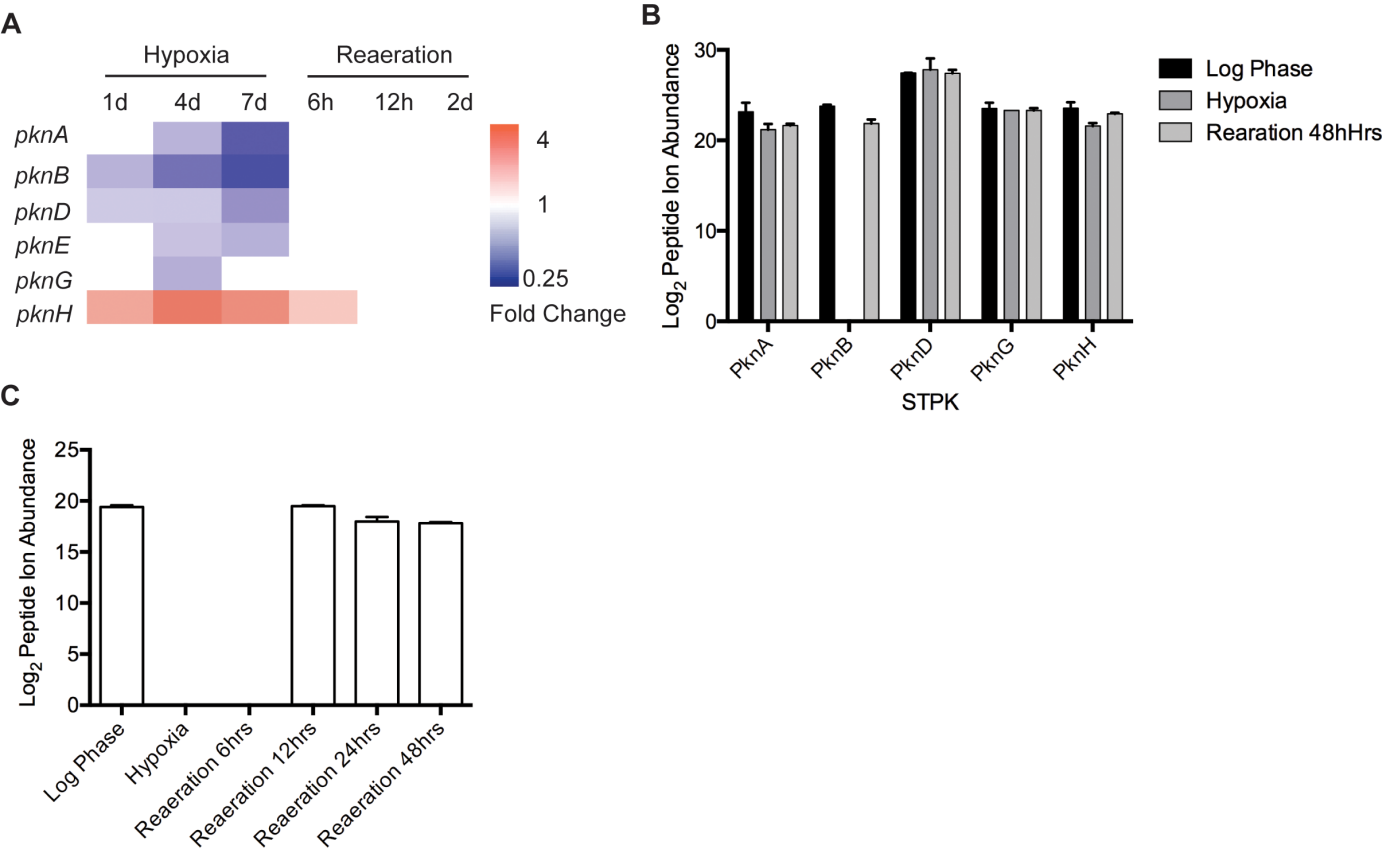
PknB transcript and protein levels change in response to oxygen. (A) Microarray analysis of STPK transcript levels during a hypoxia time course. (B) Quantitative MS analysis of STPKs in wild-type *Mtb* in log phase, day 7 of hypoxia, and day 2 of reaeration. (C) Quantitative MS analysis of PknB abundance in wild-type *Mtb*.

Because PknB is essential [23], genetic knockout is not feasible. To identify experimental conditions for manipulating PknB levels in hypoxia, we mined microarray data [21],[22] from an *Mtb* hypoxia time course to establish *pknB* transcriptional changes. *PknB* transcripts decreased ∼2-fold after exposure to hypoxia and recovered to previous levels upon reaeration, and *pknB* transcript was the most down-regulated of the STPKs measured (Figure 4A). Microarray results were confirmed by qRT-PCR in an independent analysis of an identical time course [33]. To test if protein levels changed similarly to transcript levels, we probed PknB levels by Western blot using PknB-specific rabbit IgG. PknB protein levels corresponded to transcript levels, decreasing in hypoxia and increasing to aerated levels in reaeration (Figure S5A). Since Western blotting is only semiquantitative, and because the polyclonal rabbit IgG showed significant cross-reactivity producing nonspecific background, we sought to quantitate PknB levels more reliably by mass spectrometry. We measured STPK levels by quantitative mass spectrometry, using the AMT approach as described above for ABPP analysis. *Mtb* proteomes were collected from exponentially growing cultures, day 7 hypoxic cultures, and reaerated cultures and analyzed in duplicate by mass spectrometry. Consistent with our Western analysis, PknB levels were reduced on day 7 of hypoxia, but returned to aerated levels by 12 h of reaeration (Figure 4C).

PknB levels were reduced to almost below the limit of detection on hypoxia day 7, suggesting much larger reductions of PknB than detected by Western blotting, which may be attributed to the cross-reactivity of the PknB-specific rabbit IgG. Robust peptide ion abundances were also measured for PknA, G, D, and H, but their changes were minimal compared to PknB (Figure 4A,C). The other seven STPKs not detected by mass spectrometry may be produced at low levels or may not have produced suitable peptides for detection.

These data suggest that altered PknB activity accompanies and may be causal for hypoxia-mediated transitions. These data also suggested a genetic strategy to test this hypothesis by maintaining PknB at elevated levels during hypoxia. Thus, we generated a *pknB* mutant by recombineering [34], introducing a tetracycline-inducible promoter upstream of *pknB* (Figure S4). Insertion of the tet promoter uncoupled the expression of *pknB* from the wild-type promoter. Because *pknB* is the last gene in the Rv0014c–Rv0020c operon, the promoter insertion is expected to affect only *pknB* expression. The correct insertion of the promoter cassette was confirmed by sequencing of genomic DNA. Because the tet promoter is leaky, the *tet-pknB* mutant expressed PknB at levels ∼4.5-fold higher than wild type even in the absence of anhydrotetracycline (ATc), as determined by mass spectrometry. Importantly, the uninduced *tet-pknB* mutant maintained elevated levels of PknB throughout the hypoxia time course as detected by Western blotting and mass spectrometry, allowing us to test the effects of PknB dysregulation specifically in hypoxia (Figure S5).

### Aerated Growth Is Sensitive to Changes in PknB Abundance

**Figure 5:**
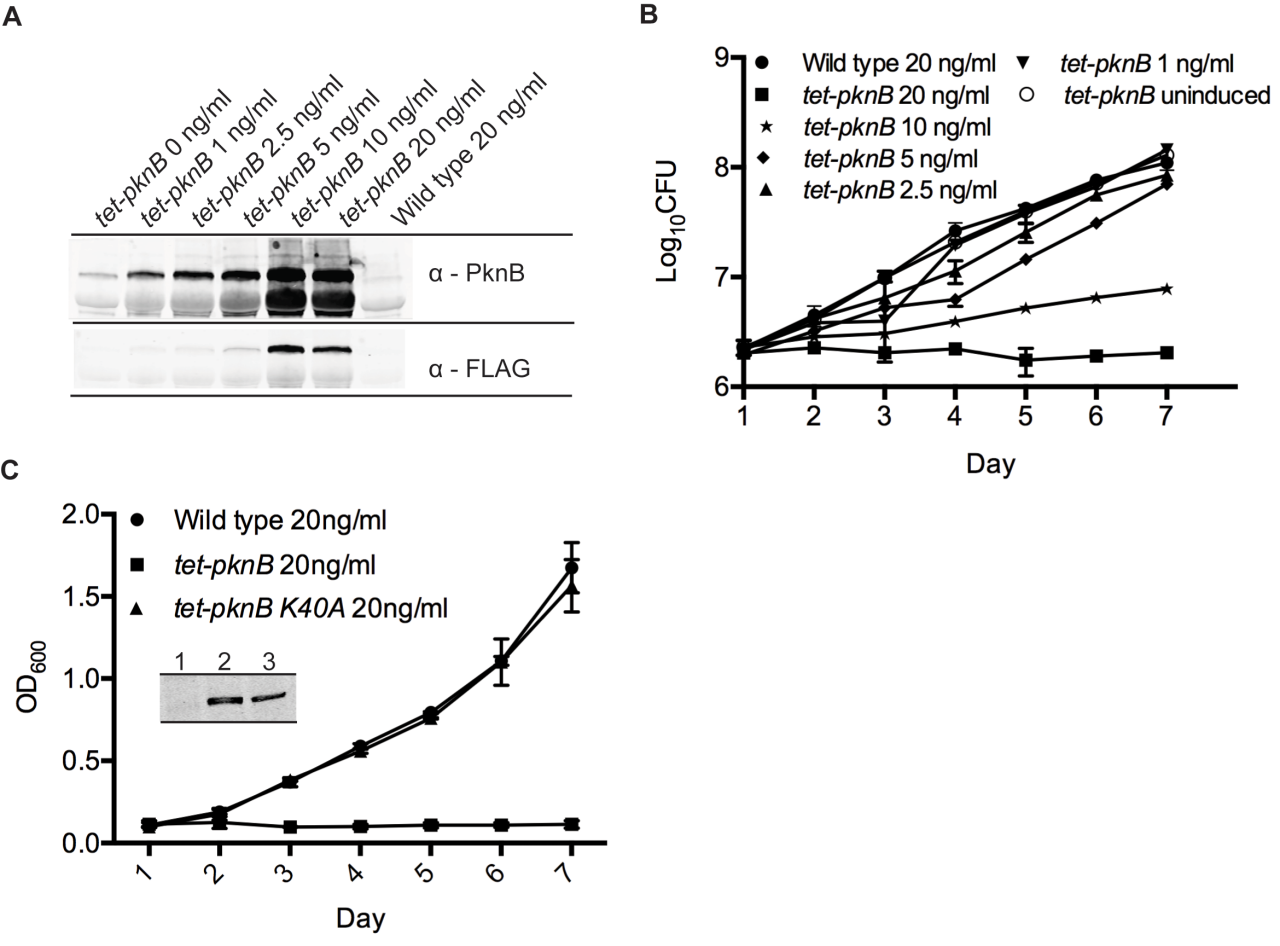
*Mtb* growth is sensitive to elevated PknB levels. (A) Overexpression of PknB by the *tet-pknB* mutant upon induction with ATc. PknB levels were determined by Western blot using PknB-specific rabbit IgG and anti-FLAG antibody. (B) Effects of PknB overexpression measured by CFU for 6 d post-ATc induction and by (C) optical density in comparison to the kinase dead mutant *tet-pknB K40A*. (Inset) PknB expression in the *tet-pknB* and *tet-pknB K40A* mutants was determined by anti-FLAG Western Blot. 1, wild type; 2, *tet-pknB*; 3, *tet-pknB K40A*, all treated with 20 ng/ml ATc. Error bars represent standard deviation.

To test if altered PknB levels affect *Mtb* growth, and to establish experimental conditions suitable for testing the role of PknB in hypoxia, we assessed the effect of increasing PknB levels using the *tet-pknB* mutant. Analysis of PknB protein levels by Western blot showed ATc-dependent induction of PknB expression (Figure 5A), which quickly resulted in a severe growth defect. ATc concentrations above 10 ng/ml caused growth arrest and cell killing as determined by CFU assay (Figure 5B). To test whether growth arrest was a result of PknB kinase activity, another PknB function, or an overexpression artifact, we generated a PknB kinase-dead overexpressing mutant, *tet-pknB K40A*. In contrast to *tet-pknB*, *tet-pknB K40A* had no effect on aerated growth at the highest levels of ATc induction (Figure 5C). Thus, the *Mtb* growth defect is indeed due to increased PknB kinase activity.

### PknB Controls Oxygen-Dependent Replication

**Figure 6:**
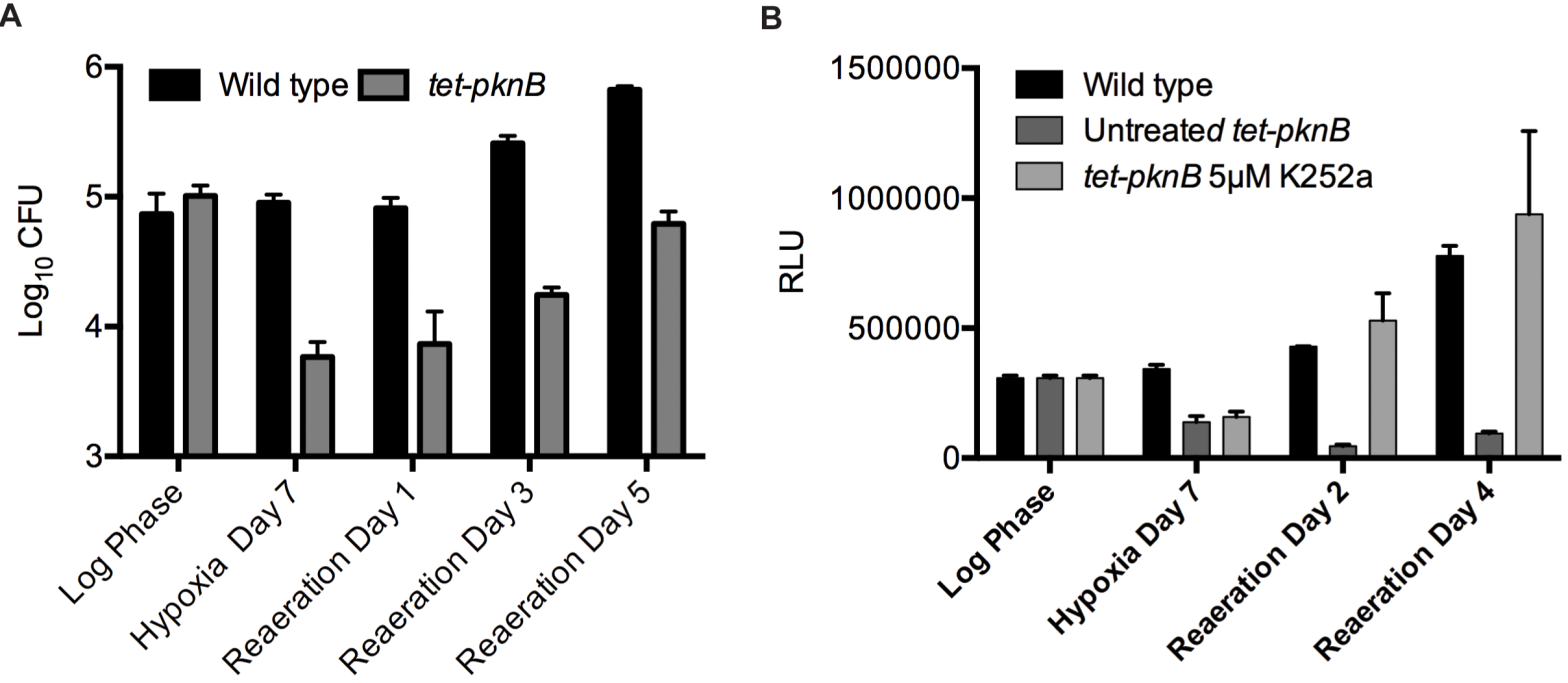
Altered PknB levels disrupt oxygen-dependent replication. Viability of (A) uninduced *tet-pknB* and (B) K252a-treated *tet-pknB* was measured by CFU and ATP levels on day 0, hypoxia day 7, and reaeration day 1 and day 3. Error bars represent standard deviation.

The uninduced *tet-pknB* mutant expressed ∼4.5-fold more PknB than wild type in aerated culture but demonstrated similar growth kinetics. Also, in contrast to the wild-type strain, the *tet-pknB* mutant expressed PknB in hypoxia (Figure S5). These properties of the *tet-pknB* mutant allowed testing for effects of PknB on hypoxic survival without affecting aerated growth. We exposed wild-type and uninduced *tet-pknB* mutant strains to hypoxia followed by reaeration. Hypoxia led to rapid growth arrest of wild-type *Mtb*, and regrowth after reaeration as previously observed [5]. During hypoxia, maintaining PknB expression at the level of aerated culture led to striking killing of the mutant. After 7 d of hypoxia, the number of *tet-pknB* was reduced 10-fold compared to wild-type *Mtb* (Figure 6A). To confirm that this *tet-pknB* survival defect was due to increased PknB activity in hypoxia, we treated *tet-pknB* with K252a to chemically revert the effects of PknB overexpression. The *tet-pknB* mutant was treated with 5 µM K252a prior to hypoxia, subjected to hypoxia for 7 d, and reaerated for 4 d. K252a-treated *tet-pknB* entered bacteriostasis in hypoxia and returned to normal growth upon reaeration, similar to wild type (Figure 6B). The ability of K252a to fully revert the PknB mediated killing in hypoxia further supports that K252a targets PknB and that increased PknB activity is responsible for *Mtb* killing in hypoxia.

To test whether PknB regulates bacteriostasis in general or specifically in response to oxygen, we tested the *tet-PknB* mutant in three other conditions encountered during infection that induce bacteriostasis: nitric oxide, low pH, and nutrient starvation. Growth and viability of the *tet-pknB* mutant were similar to wild type in all three conditions (Figure S6A, B, and C), suggesting that PknB specifically regulates growth in response to oxygen.

### *pknB* Overexpression Causes Changes in Cell Shape

**Figure 7:**
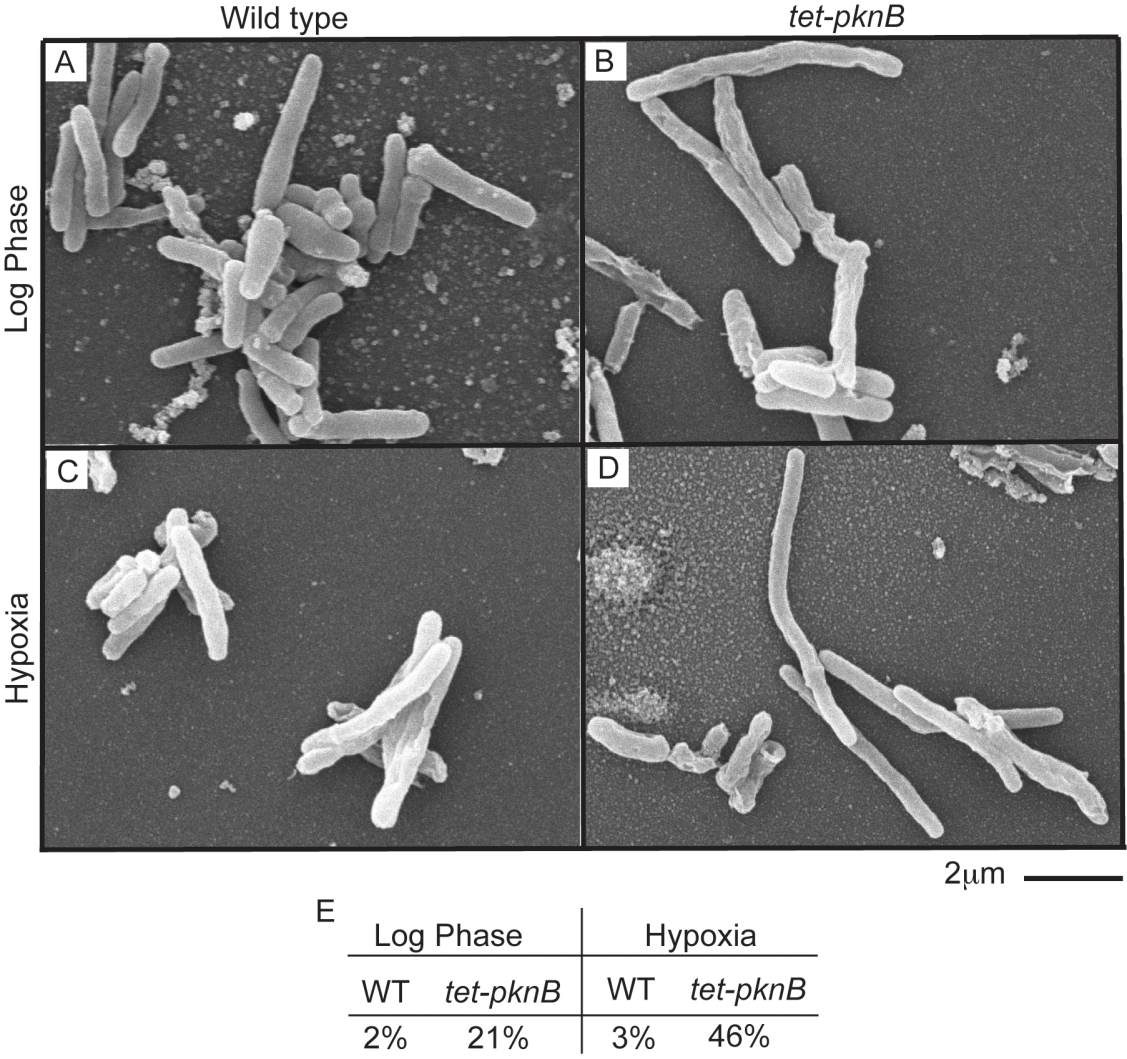
PknB overexpression leads to elongated bacilli in hypoxia. Scanning electron microscopy of (A) wild-type and (B) *tet-pknB* on day 0 (log phase), and (C) wild-type and (D) *tet-pknB* after 7 d of hypoxia. (E) The percent of elongated bacilli for each strain in log phase and hypoxia.

To test the morphological effects of elevated PknB levels, we imaged cells by scanning surface electron microscopy in aerated and hypoxic growth. Strikingly, uninduced *tet-pknB* mutants displayed an elongated morphology compared to wild-type cells. A subset of *tet-pknB* mutant cells was as much as 2-fold longer than wild-type cells, in particular in hypoxia. To quantify these differences in cell length, we determined the approximate size of >800 cells. The length of wild-type bacilli was 2–3 µm, with 2%–3% longer cells (Figure 7A, B, and E). In contrast, 20% of *tet-pknB* mutant cells in aerated and 50% in hypoxic culture were 4–6 µm long. In addition, the average length of *tet-pknB* mutant cells increased during hypoxia (Figure 7C,D).

Because overexpression of PknB in the *tet-pknB* mutant compared to wild type was only modest, and the range of PknB expression separating the phenotypes was small, higher levels of overexpression may cause more pronounced phenotypes in hypoxia. However, higher levels would also cause growth defects in aerated cultures, complicating experiments and analysis. Thus, the experimental window to test the effect of PknB in hypoxia is small, and the 10-fold reduction in CFU might underestimate the full impact of PknB on hypoxic survival.

Combined, these data establish the first link between Ser/Thr phosphosignaling and oxygen-dependent replication and identify PknB as a major regulator of this replication switch. These studies point to finely tuned regulation of growth in response to hypoxia through PknB and highlight the distinct effects of reduced and elevated PknB levels on *Mtb* growth and replication, suggesting that PknB is a highly vulnerable target for therapeutic interference with nonreplicating and reactivating *Mtb*.

## Discussion

TB pathogenesis is closely linked to *Mtb*'s ability to enter a nonreplicating state, which in turn underlies latent infection and drug tolerance. Although low oxygen tension is likely to be a major determinant of latency, little is known about the regulation of replication in response to oxygen [5]. As a consequence, only few therapeutic targets in latent, nonreplicating bacteria are known. With a genome size of ∼4.4 Mb, *Mtb* codes for a relatively large number of STPKs [4],[35], and recent phosphoproteomic data show that Ser/Thr phosphorylation varies under different growth conditions, including low oxygen tension [20], together suggesting that the STPKs regulate specialized *Mtb* adaptations. However, a causal link between *Mtb* phosphosignaling and the response to oxygen has not been established.

The 11 *Mtb* STPKs may have redundant function, and two are essential, complicating genetic approaches. To overcome this hurdle, we used a chemical biology approach to test for a causal role of Ser/Thr phosphosignaling in the response to oxygen. While the broad kinase inhibitor staurosporine was a useful tool to reveal a functional link between STPKs and hypoxia survival, its binding promiscuity precluded direct identification of the responsible kinase(s). However, using staurosporine in combination with competitive ABPP allowed us to identify the STPK(s) responsible for the staurosporine phenotype. This approach has several advantages over standard loss-of-function genetics. With 11 STPKs and potential redundancy of their functions, genetic approaches are limited. The use of the generic kinase inhibitor staurosporine in combination with ABPP, in contrast, allowed for broad screening and specific identification of STPKs that mediate the staurosporine phenotype. This approach identified three STPK staurosporine targets (PknB, PknD, and PknF), providing a tractable number for genetic determination of the individual contributions of STPKs to oxygen-dependent replication. In addition, these data provide a comprehensive staurosporine binding profile in the *Mtb* proteome. Although we used a broad specificity kinase inhibitor in our studies, our MS analysis shows that staurosporine bound only a subset of STPKs. Thus, although our experiments unequivocally link PknB with oxygen-dependent growth transitions, we did not sample all STPKs, and other STPKs not sensitive to staurosporine might also contribute to hypoxic adaptations.

Changing *pknB* levels in either direction showed large effects on cell growth even in aerated culture, and the experimental window to test *pknB* effects in hypoxia was small. To determine the best genetic approach for capturing the specific contribution of *pknB* to oxygen-dependent replication, we first established the transcript and protein abundance of PknB in aerated, hypoxic, and reaerated culture. Quantitative mass spectrometry showed a dramatic decrease in PknB protein levels in hypoxia. This reduction cannot be explained by the relatively modest changes in *pknB* transcript levels, but points towards specific PknB degradation. While many STPKs are regulated by reversible phosphorylation [36], PknB appears to be regulated also on the level of protein abundance, a STPK regulatory mechanism that implies the contribution of specific proteolysis in the PknB regulatory pathway. In addition, these experiments suggested a genetic approach that allows for hypoxia-specific *pknB* expression above wild-type levels specifically during hypoxia.

**Figure 8:**
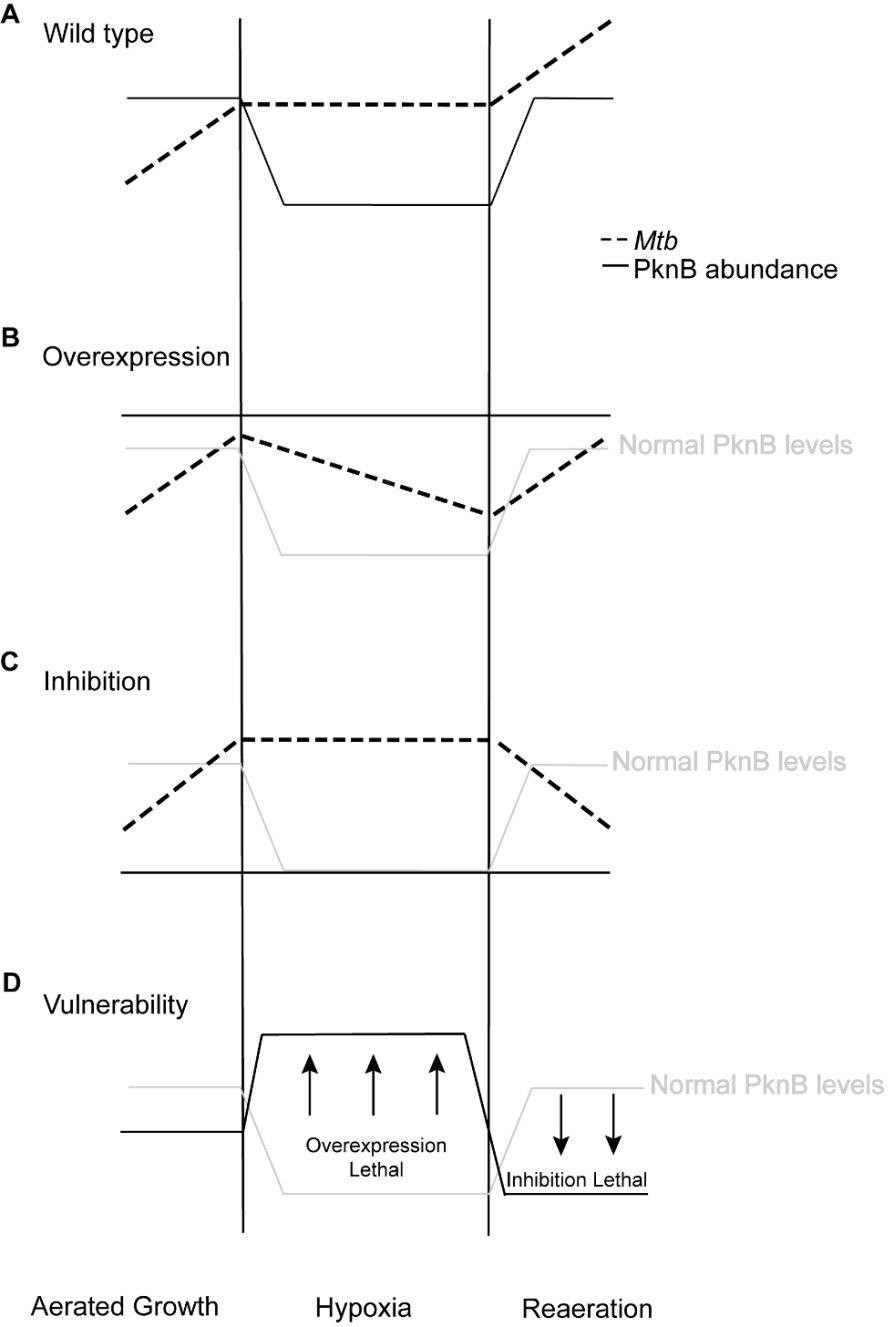
Effects of PknB dysregulation in hypoxia and reaeration. (A) *Mtb* growth (dashed line) is regulated by PknB (solid line) in response to oxygen tension. Under low oxygen conditions in hypoxia, PknB activity is decreased to facilitate growth arrest, but returns upon reaeration to support regrowth. PknB dysregulation by (B) overexpression or (C) inhibition interferes with survival at different stages. (D) *Mtb* growth is most vulnerable to PknB targeting in hypoxia and reaeration. Elevated PknB levels in hypoxia and reduced levels in reaeration are most detrimental.

Our data show that PknB levels are carefully calibrated during hypoxia and reaeration, consistent with a role of PknB in replication, which is essential in reaeration but detrimental during hypoxia (Figure 8). Changes in PknB levels have dramatic effects on cell survival that are also reflected by dramatic changes in cell morphology. The highly elongated cells resulting from PknB overexpression in aerated growth and particularly in hypoxia point to a major role of PknB in cell elongation and/or division. Elevated PknB levels drive cell elongation, but elongation appears to be decoupled from cell division, particularly in hypoxia. A previous study demonstrated widened and bulging bacilli upon overexpression of *Mtb pknB* in *M. smegmatis* and *M. bovis* [18]. This difference in phenotype from the one described here may be a result of cross-species expression in the former study.

PknB specifically regulates growth in response to oxygen, as other stresses encountered *in vivo* did not affect the survival of the *tet-pknB* mutant. How does PknB sense changing oxygen tension? The four extracellular penicillin binding protein and serine/threonine kinase associated (PASTA) domains of PknB do not suggest direct oxygen sensing [37]. The PASTA sensor domains appear to regulate localization of PknB to the cell poles and septum through binding of muropeptides [38]. We propose that PknB indirectly senses shifting oxygen tension, for example through phosphorylation by another STPK or a two-component system. Crosstalk between STPKs and between STPKs and two component systems in *Mtb* has previously been shown [18],[32]. For example, the STPK PknH phosphorylates DosR, providing a possible connection between STPK and two-component system signaling in response to hypoxia. However, we did not detect an effect of this PknH on survival in hypoxia. These data likely reflect the limited role of DosR in the response to hypoxia [21]. Alternatively, an oxygen sensor might affect peptidoglycan levels or structure that is sensed through the PknB PASTA domains and provides appropriate localization of PknB. The role of PknB in adapting to changing oxygen and replication invites a comparison to the role of another bacterial STPK carrying PASTA domains, the *B. subtilis* STPK PrkC. In *B. subtilis*, PrkC controls the germination of spores in response to peptidoglycan fragments and free muropeptides [39]. The similarities between stress response programs between these two organisms suggest conservation of linked sensing of oxygen and peptidoglycan signals across different phyla. The functional consequences of PknB are ultimately defined by its substrates. By identifying the first direct link between Ser/Thr phosphosignaling and latency-related adaptations, this study provides evidence that the downstream hypoxia effectors are at least in part phosphoproteins and specific PknB substrates, suggesting new experimental approaches to identify these targets.

This study also provides a first measure of PknB's vulnerability, an important metric for drug targets. PknB is currently viewed as a viable drug target in actively growing *Mtb* [40]–[42]. Our data show that PknB levels are dramatically reduced during hypoxia, requiring PknB synthesis in reaeration. As a result, reduced target levels decrease the minimal inhibitory concentration for PknB inhibitors during reaeration, and by extension in reactivation. Prevention of reactivation could have significant benefit, especially in settings with high HIV burdens and the associated high rates of reactivating disease. In contrast to reactivating disease, our data suggest that an effective strategy to kill nonreplicating *Mtb* would be through PknB activation rather than inhibition. Chemical activation of STPKs is not well understood, and common mechanisms of chemical STPK activation are not known. Yet the concept of therapeutic STPK activation is not without precedent. The lactone compound bryostatin is under active development as a protein kinase C activator [43], and bryostatin leads to PrkC activation and the exit from dormancy in *B. subtilis* [39]. Similarly, PknB activation could drive PknB activity during nonreplication, leading to growth signals at a time when growth is deadly.

Together, our data show that Ser/Thr phosphosignaling regulates replication in response to oxygen. We identify PknB as a critical regulator of oxygen-dependent replication, and by extension of latency and the progression to active disease. By reacting to oxygen changes, PknB triggers adaptations to low oxygen stress and is itself down-regulated in the effort to cease growth. Even small increases in PknB levels during nonreplication lead to bacterial killing. As growth resumes upon reactivation, PknB activity is required and loss of activity at this stage also leads to killing. In this way, PknB provides a potential, highly vulnerable drug target to target *Mtb* at every stage of its life cycle.

## Materials and Methods

### Strains, Culture Conditions, and Hypoxic Model

Frozen aliquots of each strain were thawed and expanded to generate working stocks before experiments. H37Rv (ATCC 27294), *tn:pknD*, *tn:pknF*, *tn:pknH*, and *tet-pknB* were grown at 37°C in Middlebrook 7H9 supplemented with OADC and 0.05% Tween 80 in rolling culture. The hypoxia time course was carried out as previously described [21]. Briefly, cultures were grown to reach early log phase (OD_600_ 0.1–0.3) and placed in 250 ml spinner flasks to stir at 60 RPM with low oxygen gas (0.2% O_2_ balanced with N_2_) flowing over the culture for 7 d. For reaeration, cultures were returned to normoxia and grown for an additional 4 d to fully reaerate.

The *Mtb tet-pknB* mutant strain was created by inserting the tetracycline regulation elements *tetO* and *tetR* along with the hygromycin resistance gene 5′ to the *pknB* start codon, and a FLAG tag at the *pknB* N terminus (Figure S5). *Mtb* H37Rv genomic DNA was used as the template to amplify the 500 bp regions flanking the 3′ end of the *pknA* gene and the 5′ end of the *pknB* gene. The PCR fragments were then cloned into vector pRPK339. Amplification of the recombineering substrate was performed by high fidelity PCR. Electroporation was performed as described previously [44]. Colonies were selected on 7H10 plates with hygromycin. Resistant colonies were grown to an OD_600_ of 1, genomic DNA prepared, and screened for correct insertion by DNA sequencing of genomic DNA.

To induce *pknB* expression in the *tet-pknB* mutant, cultures were grown to an OD_600_ of 0.1, and anhydrotetracycline was added at concentrations from 1–20 ng/ml. Growth was monitored by OD_600_ for 6 d postinduction.

### Nitric Oxide, pH, and Starvation Experiments

For nitric oxide experiments, liquid cultures were grown to early log phase (OD 0.1–0.3) and then treated daily with 100 µM DETA-NO (Sigma) for 4 d [45]. On the 5th day, cultures were centrifuged at 4,000× g for 5 min and the medium replaced with 7H9 without DETA-NO. Growth was measured daily by OD_600_ and CFU for a total of 7 d.

For pH experiments, cultures were grown to early log phase (OD 0.1–0.3) and then centrifuged at 4,000× g for 5 min. Medium was replaced with 7H9 at pH 4.5 [46]. Growth was measured daily by OD_600_ and CFU for 5 d. For starvation experiments, 7H9 medium was replaced with 1× PBS. Growth was measured by OD_600_ and CFU for 4 wk.

### RNA Isolation and Microarrays

RNA was isolated as previously described [5]. Briefly, cultures were grown to an optical density at 600 nm of 0.1–0.3. We centrifuged 25 ml of culture at 4,000× g for 5 min and pellets were resuspended in 1 ml of Trizol (Gibco-BRL). The sample was lysed by bead beating 3 times at 5.5 m/s for 30-s intervals, with cooling between intervals. Lysis was followed by centrifugation for 1 min at maximum speed. Supernatant was transferred to a Phase-loc tube (5 Prime) containing 300 µl of chloroform∶isoamyl alchohol (1∶24) and centrifuged for 5 min at maximum speed. The aqueous layer was transferred to 1.5 ml tubes containing 270 µl of isopropanol and 270 µl of high salt solution (0.8 M Na Citrate, 1.2 M NaCl) and precipitated overnight at 4°C. The samples were then centrifuged for 10 min at 14,000 g at 4°C, supernatant was decanted, and RNA purification was performed as described in the Qiagen RNeasy Purification Kit protocol. Microarray data were obtained from a previously published study [24].

### Cell Lysate Preparation

Cell lysate was prepared from 100 ml of culture. Bacteria were centrifuged at 4,000× g, and pellets resuspended in 500 µl of lysis buffer (1× PBS, 0.5% SDS, NaF, β-glycerophosphate, and protease inhibitor cocktail (Sigma)). Samples were lysed by bead beating three times at 5.5 m/s for 30-s intervals, with cooling on ice between intervals. Samples were centrifuged at 14,000× g for 2 min, and supernatant was sterile filtered twice with 0.2 µM filters. Cell lysates were stored at −80°C.

### Western Blotting

We separated 20 µg of log phase, hypoxic, and reaerated cell lysates by 4%–12% SDS-PAGE. Protein was transferred to nitrocellulose membrane using a semidry transfer system. Membranes were blocked with 3% BSA in 1× PBS/0.1% Tween 20 for 1 h and incubated overnight at 4°C with either 1∶500 of antiphosphoserine and antiphosphothreonine antibody (Millipore), 1∶2,000 of rabbit anti-PknB IgG purified from rabbit serum on protein G sepharose, or 1∶1,000 M2 anti-FLAG antibody. Membranes were washed in 1× PBS/0.1% Tween 20 and incubated for 1 h at room temperature with either 1∶15,000 anti-rabbit or anti-mouse fluorophore-conjugated secondary antibody (LI-COR Biosciences). Membranes were washed in 1× PBS – 0.1% Tween 20 and imaged using an Odyssey imager (LI-COR Biosciences).

### Competitive ABPP

Detergent-solubilized lysate (1 mg per sample) was incubated with 500 µM staurosporine (BioSciences) for 30 min at 37°C. Then, treated and untreated samples were labeled with 20 µM ATP-ABP for 1 h at 37°C, followed by treatment with biotin-azide (36 µM), TCEP (2.5 mM), TBTA (250 µM), and CuSO_4_ (0.50 mM). The samples were vortexed and incubated at room temperature in the dark for 1.5 h. Probe-labeled proteins were enriched on streptavidin agarose resin, reduced with TCEP, and alkylated with iodoacetamide. Finally, proteins were digested on-resin with trypsin, and the resulting peptides collected for LC-MS analysis.

### LC-MS Analysis of Competitive ABPP and Global Proteomics

Quantitative proteomics data were generated and analyzed using the AMT tag approach [47]. Tryptic peptides from labeled and unlabeled samples were collected for LC-MS analysis as described previously [28] and analyzed using a LTQ-Orbitrap Velos (Thermo Fisher Scientific) MS interfaced with a reverse phase HPLC system. MS/MS spectra were searched using the SEQUEST algorithm (V27, revision 12) [48] against the publicly available H37Rv translated genome sequence, and re-scored using the MS-GF approach [49]. Peptides at least six amino acids in length with MS-GF scores ≤1E-10, which corresponds to an estimated false discovery rate <1% at the peptide level, were used to generate an AMT tag database. Mass and elution time features were identified and matched with VIPER to peptides stored in the AMT tag database within mass measurement accuracy and elution time accuracy cutoffs of <2 ppm and <2%, respectively. The measured abundance value for a particular peptide was determined by integrating the area under each LC-MS peak for the detected feature matching to that peptide. Matched features were then filtered on a false discovery rate of ≤5% using STAC [49]. Peptide abundance measurements in technical replicates were scaled and normalized to the data set with the least information using linear regression in DAnTE [50], then rolled up to proteins using RRollup [50]. A minimum of five peptides was required for the Grubb's test, with a *p* value cutoff of 0.05. Only peptides unique in identifying a single protein were utilized to estimate protein abundances. Additionally, proteins represented by <2 unique peptides were excluded. If peptides for a given protein were not measured in at least half the replicates for a given sample type, the protein was excluded from further analysis.

### Inhibitor Experiments

H37Rv cultures were grown for three doublings and diluted to an optical density of 0.05–0.1 at 600 nm before treatment with inhibitor. Growth was determined daily by optical density for 7 d. For hypoxia inhibitor experiments, cultures were grown for three doublings to reach early log phase, placed in 250 ml spinner flasks, and inhibitor was added. Cultures were immediately subjected to low oxygen gas (0.2% O_2_ balanced with N_2_) for 7 d, followed by normoxia for 4 d to reaerate. Samples were taken at day 0, day 7 of hypoxia, and every other day of reaeration to measure viability by CFUs and BacTiter-Glo (Promega), as described in the manufacturer's protocol.

## Supporting Information

Data S1 **Staurosporine binding profile in the** ***Mtb*** **proteome.** Rv numbers, fold change between staurosporine treated and untreated samples, and peptide ion abundance as log_2_ are shown.
(XLSX)

Figure S1 **Inhibition by staurosporine compromises viability in reaeration.** ATP levels were measured using the BTG assay on day 1 of reaeration. Error bars represent standard deviation.
(TIF)

Figure S2 **PknD, PknF, and PknH do not have a role in oxygen-dependent replication.** (A) Growth kinetics of *tn:pknD* and *tn:pknF* were similar to wild-type. Viability in hypoxia and reaeration, determined by CFU, for (B) *tn:pknD*, (C) *tn:pknF*, and (D) *tn:pknH* was also comparable to wild type. Error bars represent standard deviation.
(TIF)

Figure S3 **K252a inhibition compromises viability in reaeration.** ATP levels were measured using the BTG assay on day 1 of reaeration. Error bars represent standard deviation.
(TIF)

Figure S4 **Schematic of the** ***tet-pknB*** **overexpression mutant.** The recombineering substrate consisted of a hygromycin cassette (*hyg*), the tetracycline promoter elements (*tetR* and *tetO*), and an N-terminal FLAG tag (F), flanked by 500 base pairs of homology to the 3′ end of the *pknA* gene (796–1296) and the 5′ end of the *pknB* gene (1–500). Electroporation into mycobacteria was performed as described previously [44].
(TIF)

Figure S5 **Uninduced** ***tet-pknB*** **overexpresses PknB in hypoxia.** PknB protein levels measured by (A) Western blot using PknB-specific rabbit IgG and (B) mass spectrometry in wild-type and *tet-pknB* cell lysate during a hypoxia time course.
(TIF)

Figure S6 **PknB mediates an oxygen-specific replication switch.** The *tet-pknB* mutant was exposed to (A) nitric oxide daily for 4 d, (B) pH 4.5 for 5 d, and (C) nutrient starvation for 4 wk. CFUs were compared by two-way ANOVA and not significantly different between wild-type and *tet-pknB*. Error bars represent standard deviation.
(TIF)

## Abbreviations

ABPP: activity-based protein profiling
AMT: accurate mass and time
ATc: anhydrotetracycline
ATP-ABP: ATP activity-based probe
CFU: colony forming units
*Mtb*: *Mycobacterium tuberculosis*
PASTA: penicillin binding protein and serine/threonine kinase associated
STPK: serine/threonine protein kinase
TB: tuberculosis
