# Supplementary figures and images for: Mycobacterium tuberculosis Ser/Thr Protein Kinase B Mediates an Oxygen-Dependent Replication Switch
Source: PLoS Biol. 2014 Jan 7;12(1):e1001746. doi: 10.1371/journal.pbio.1001746 (PMC3883633; doi:10.1371/journal.pbio.1001746)

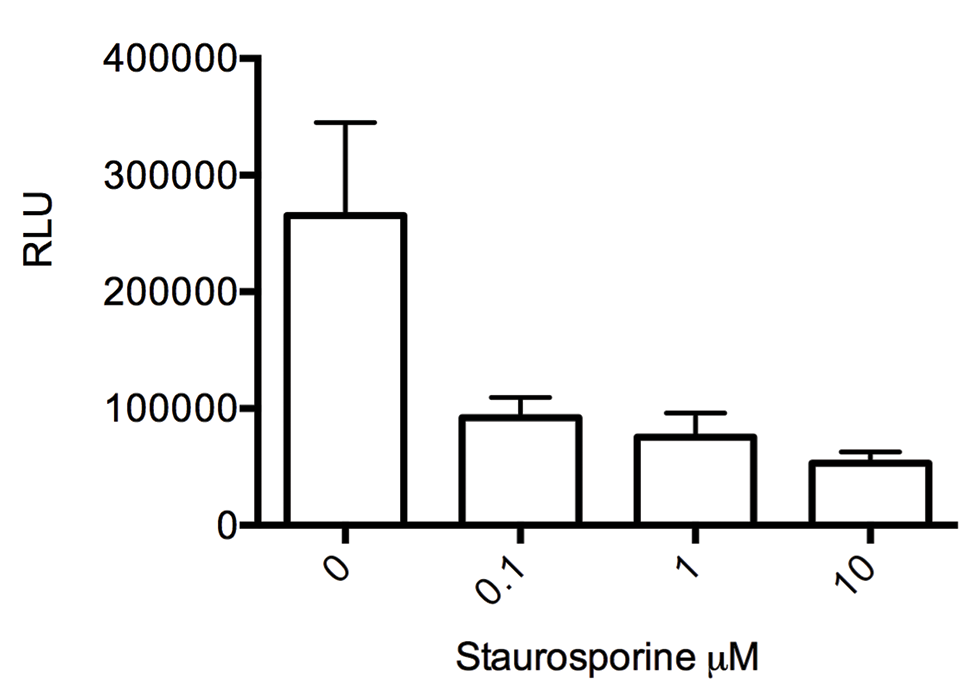

Supplement: Figure S1 — Inhibition by staurosporine compromises viability in reaeration. ATP levels were measured using the BTG assay on day 1 of reaeration. Error bars represent standard deviation. (TIF) [file pbio.1001746.s002.tif]

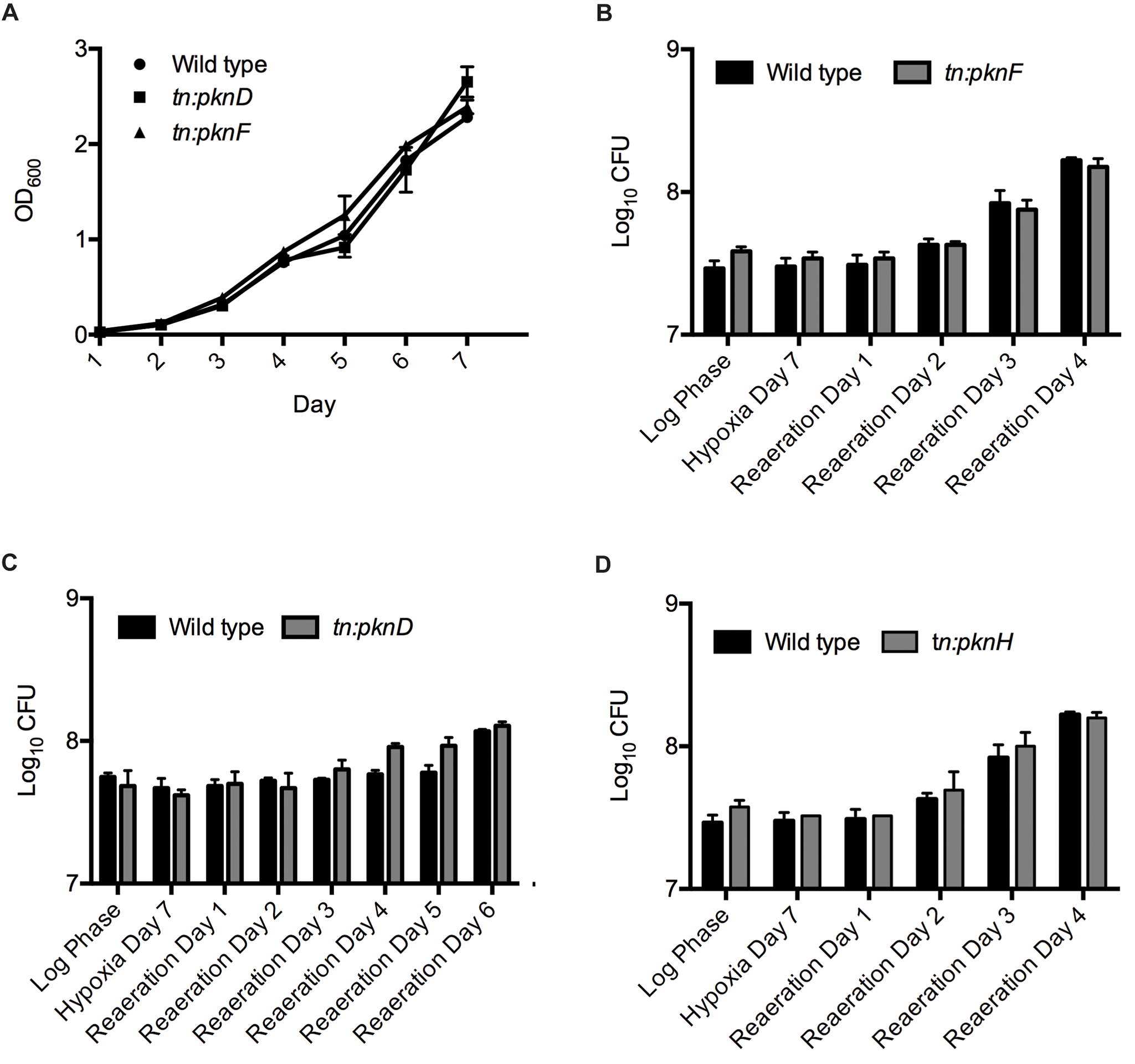

Supplement: Figure S2 — PknD, PknF, and PknH do not have a role in oxygen-dependent replication. (A) Growth kinetics of tn:pknD and tn:pknF were similar to wild-type. Viability in hypoxia and reaeration, determined by CFU, for (B) tn:pknD, (C) tn:pknF, and (D) tn:pknH was also comparable to wild type. Error bars represent standard deviation. (TIF) [file pbio.1001746.s003.tif]

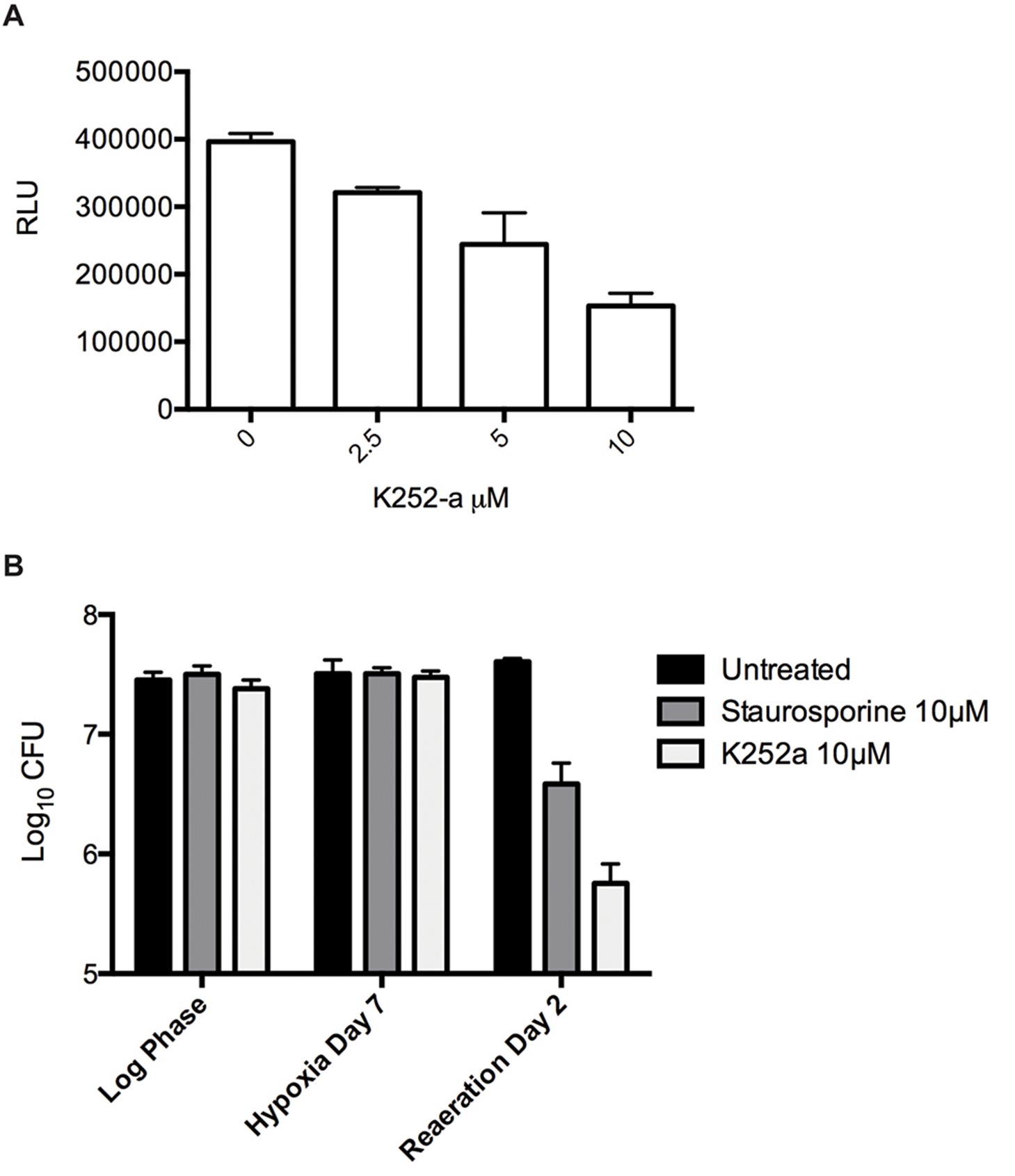

Supplement: Figure S3 — K252a inhibition compromises viability in reaeration. ATP levels were measured using the BTG assay on day 1 of reaeration. Error bars represent standard deviation. (TIF) [file pbio.1001746.s004.tif]

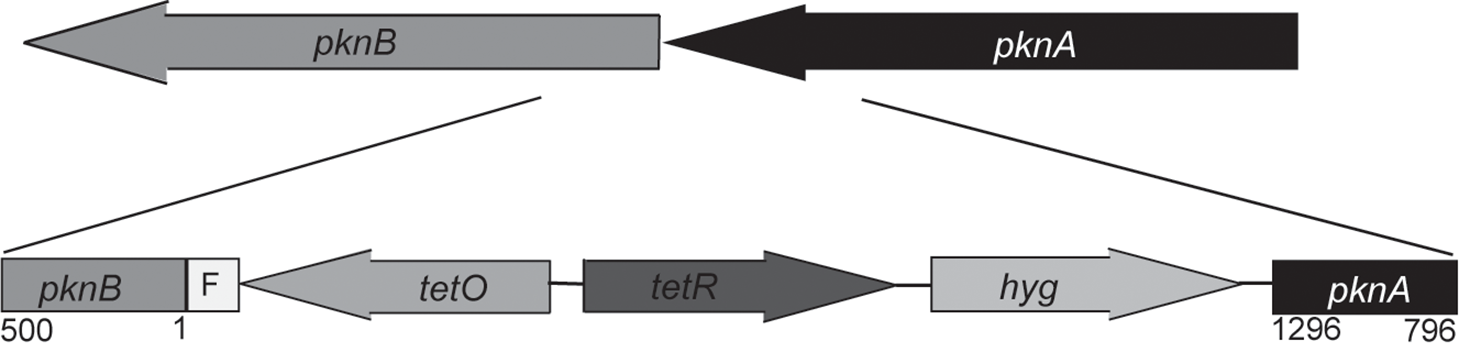

Supplement: Figure S4 — Schematic of the tet-pknB overexpression mutant. The recombineering substrate consisted of a hygromycin cassette (hyg), the tetracycline promoter elements (tetR and tetO), and an N-terminal FLAG tag (F), flanked by 500 base pairs of homology to the 3′ end of the pknA gene (796–1296) and the 5′ end of the pknB gene (1–500). Electroporation into mycobacteria was performed as described previously [44]. (TIF) [file pbio.1001746.s005.tif]

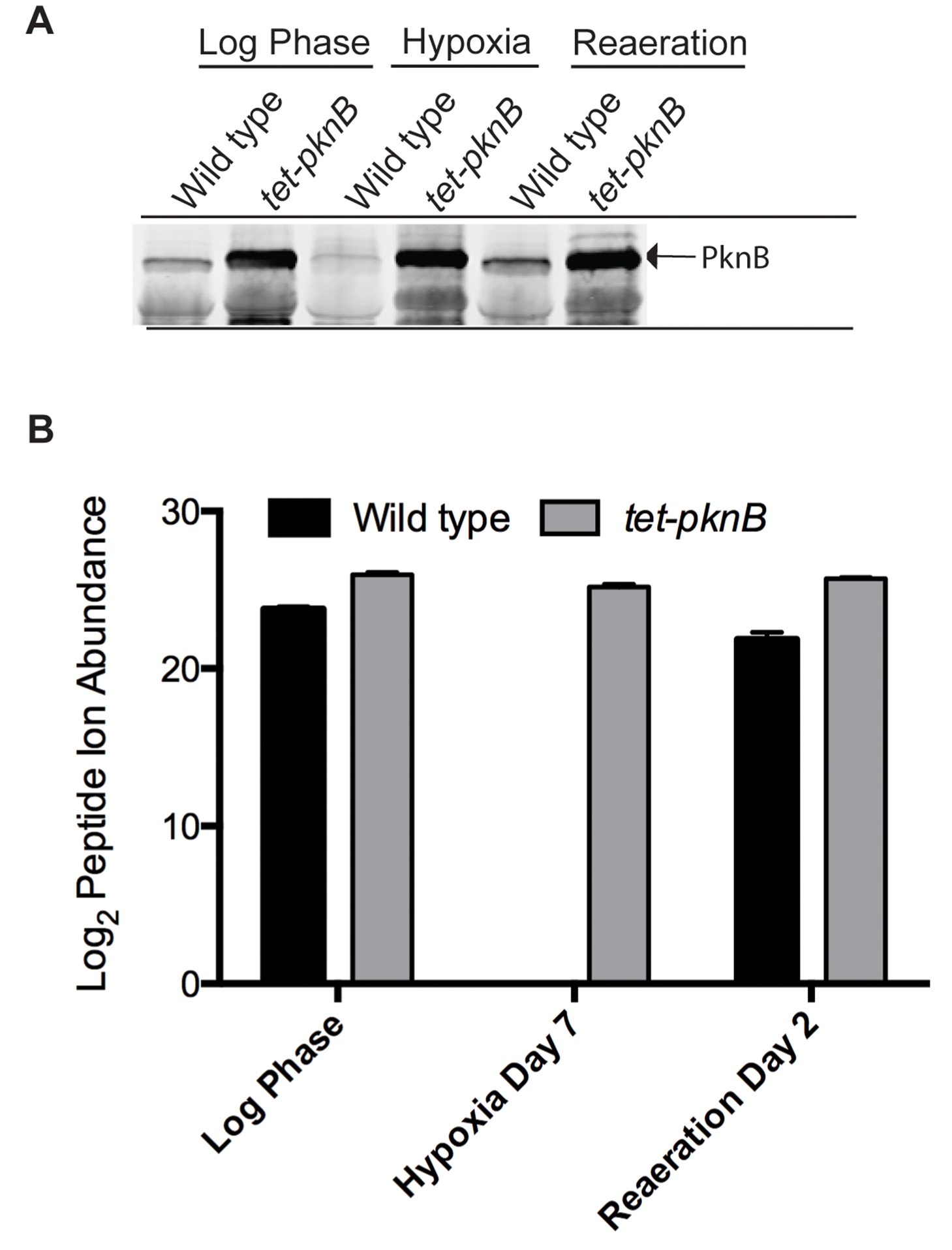

Supplement: Figure S5 — Uninduced tet-pknB overexpresses PknB in hypoxia. PknB protein levels measured by (A) Western blot using PknB-specific rabbit IgG and (B) mass spectrometry in wild-type and tet-pknB cell lysate during a hypoxia time course. (TIF) [file pbio.1001746.s006.tif]

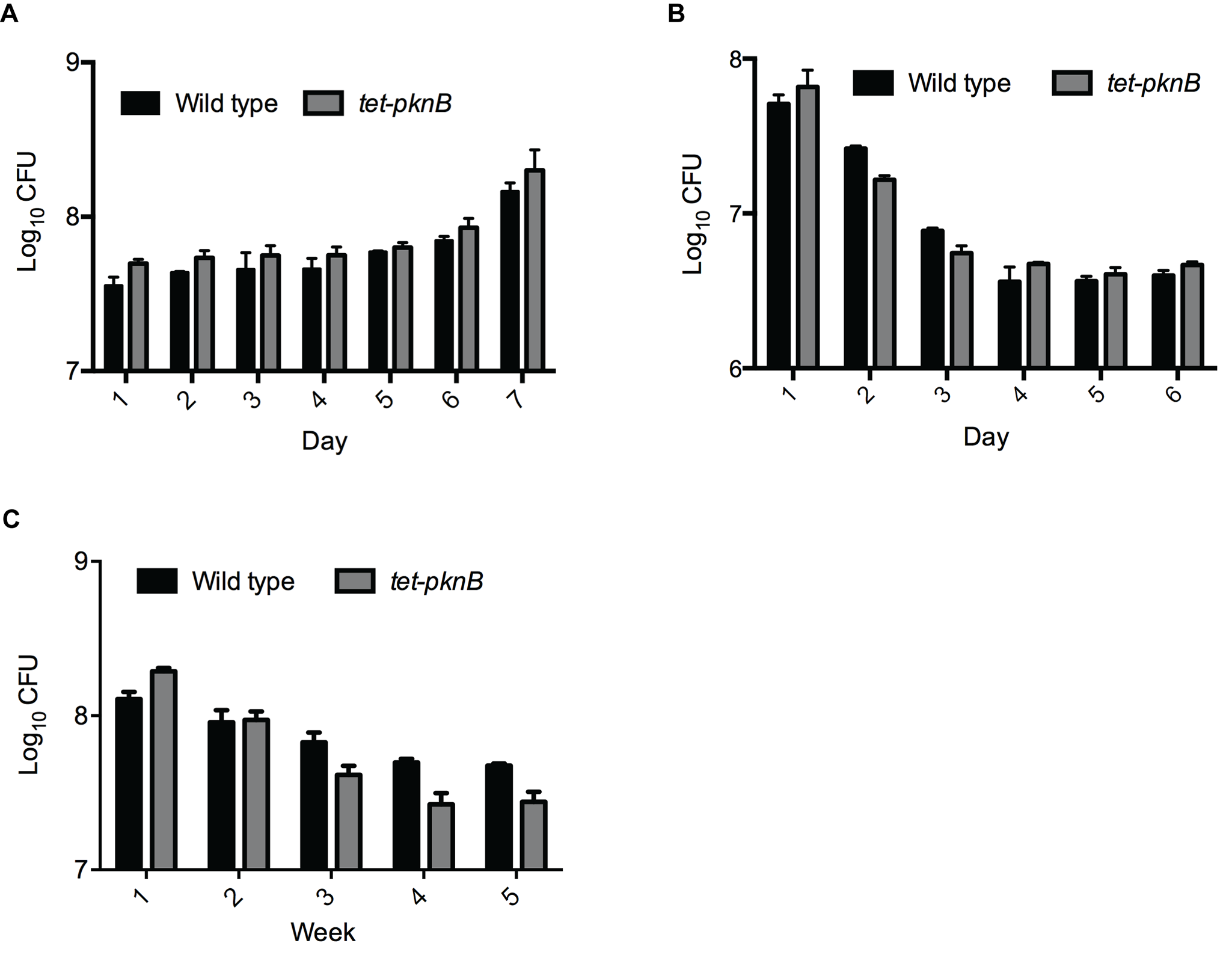

Supplement: Figure S6 — PknB mediates an oxygen-specific replication switch. The tet-pknB mutant was exposed to (A) nitric oxide daily for 4 d, (B) pH 4.5 for 5 d, and (C) nutrient starvation for 4 wk. CFUs were compared by two-way ANOVA and not significantly different between wild-type and tet-pknB. Error bars represent standard deviation. (TIF) [file pbio.1001746.s007.tif]
